# Sense of coherence and self-concept in Lynch syndrome

**DOI:** 10.1186/1897-4287-11-7

**Published:** 2013-07-05

**Authors:** Helle Vendel Petersen, Steen Ladelund, Christina Carlsson, Mef Nilbert

**Affiliations:** 1 Department of Oncology, Institute of Clinical Sciences, Lund University, Lund, Sweden; 2 The Danish HNPCC-register, Clinical Research Centre, Copenhagen University Hospital, Hvidovre Kettegård allé 30, 2650, Hvidovre, Denmark

**Keywords:** Hereditary cancer, HNPCC, Psychosocial support, Comparison with general population, Age differences

## Abstract

**Objective:**

Most individuals who learn about hereditary cancer manage well, but identification of subgroups who find this knowledge burdening would allow psychosocial intervention. The objective of the study was to assess sense of coherence (SOC) in individuals with Lynch syndrome with comparison to a general population and correlation to self-concept.

**Methods:**

A total of 345 individuals with Lynch syndrome completed the 13-item SOC scale and the 20-item Lynch syndrome self-concept scale. SOC scores were compared to a general Danish population and were correlated to self-concept estimates in individuals with Lynch syndrome. Characteristics of subgroups with adverse scores were described.

**Results:**

Individuals with Lynch syndrome reported SOC scores similar to the general population. SOC and self-concept correlated well with a correlation coefficient of −0.51. Subsets with convergent and divergent scores, which may reflect different effects from knowledge about hereditary cancer, were identified.

**Conclusion:**

Individuals with Lynch syndrome report SOC scores similar to the general population. SOC and self-concept correlate well but allow identification of subset who report adverse outcome and may be relevant for targeted intervention.

## Introduction

Genetic diagnostics is available for an increasing number of hereditary syndromes, which implies that a growing number of families live with knowledge about hereditary cancer. Lynch syndrome is a multi-cancer syndrome caused by inherited DNA mismatch-repair gene mutations that confer particularly high risks for colorectal cancer and gynaecological cancer. Genetic counselling provides information about the trait, possibilities for genetic testing and recommendations for cancer-preventive surveillance programs. In Lynch syndrome, genetic diagnostics is requested by most family members at risk, and is beneficial since colonoscopic surveillance effectively reduces morbidity and mortality from colorectal cancer [1-5].

Individuals in families with hereditary cancer have often experienced death from cancer in close relatives and can be hypothesized to be burdened from these experiences [6]. Studies that have addressed the psychological well-being in individuals at increased risk of cancer have primarily focused on the time around genetic counselling and have in the majority of individuals demonstrated temporarily increased levels of distress and anxiety with return to base-line 6–12 months after genetic testing [7-10]. A subset of 5-10% of the individuals, however, report persistent distress related to their test result [9,11,12]. A history of psychosocial problems or depression has been found to correlate with increased anxiety and distress after genetic testing [12-14]. Also, coping style, external and internal factors such as personal resources, risk perception, social networks, interaction with health care providers and access to psychosocial support have been found to influence the ability to handle knowledge about an increased risk of cancer [15,16]. Though few studies have evaluated the long-term consequences of living with a high risk of cancer, a number of issues have been identified as challenging, e.g. risk perception, colonoscopies, informing children and family communication [9,12,15,17-20].

We assessed sense of coherence (SOC) and self-concept in carriers of Lynch syndrome. SOC and self-concept are related in that SOC expresses a person’s perceived ability to handle difficult situations, whereas self-concept reflects the impact specific situations or circumstances may have on a person’s self. Perceptions of the self are considered powerful determinants of behaviour. Through an internal system of knowledge structures, experiences constitute the cognitive foundation of purposive thoughts and actions. Self-concept relates to how people think about and evaluate themselves in specific situations [21]. Individuals with a positive self-concept are more apt at enduring stressful situations as opposed to those with a more negative perception of the self [21]. Self-concept scales for different types of hereditary cancer have been developed [16,22-24]. The Lynch syndrome self-concept scale contains two subscales related to stigma and vulnerability and to gastrointestinal-related anxiety. The scale has shown high convergent validity and promising psychometric characteristics in Western populations [24-26]. SOC represents a global estimate of how individuals perceive and handle difficult situations and is based on the three nuclear components comprehensibility, manageability and meaningfulness [27-29]. The SOC scale has been validated in different subgroups with satisfactory performance [29-31]. SOC is positively correlated to mental health and strong SOC has been found to correlate with low depressive mode, high self-esteem and an optimistic life orientation [27,32-34]. Individuals with strong SOC are estimated to react more appropriately to stressful situations by using relevant personal coping strategies. Women diagnosed with breast cancer, who reported strong SOC, experience fewer stressful events and reported better health status [35,36]. Consequently, weak SOC has been shown to correlate with anxiety and depression [31,37]. We aimed to determine SOC in the Lynch syndrome population with comparison to the general population and to correlate SOC and self-concept in individuals with Lynch syndrome.

## Method

We used the national Danish hereditary nonpolyposis colorectal cancer (HNPCC) register to identify Lynch syndrome mutation carriers. Data on SOC and self-concept were collected at two occasions; from 200 individuals with verified mutations for Lynch syndrome in Western Denmark in 2009 (as part of a nation-wide self-concept study [26]) and from 145 recently identified mutation carriers (data not previously presented).

The Lynch syndrome self-concept scale contains 20 statements related to gastrointestinal anxiety and stigma-vulnerability with scores ranging from 20–140 on the total scale and from 5–35 and 15–105 on the bowel symptom-related anxiety subscale and the stigma and vulnerability subscale respectively. The lower scores, the less impact on self-concept [24]. SOC was assessed using the 13-item scale with scores ranging from 13–91 with higher scores representing stronger SOC [28-30,34]. Aggregated SOC results from a general Danish population collected within the Danish Longitudinal Health Behaviour study in 1994 were used for comparison [38].

We used the same cut-off values for weak (<63), under average (63–73), over average (74–79) and strong (> 79) in the Lynch syndrome cohort as in the general population to allow for direct comparison. Matching age groups were generated with 7 Lynch syndrome groups with age group 1 (age <25, n = 14), age group 2 (age 25–34, n = 58), age group 3 (age 35–49, n = 197), age group 4 (age 50–59, n = 75), age group 5 (age 60 –69, n = 49), age group 6 (age 70 –79, n = 22) and age group 7 (age >80, n = 5, data not shown due to the low number of individuals). The data from the general-population consisted of the 5 age cohorts born 1975 (n = 663), 1965 (n = 563), 1940 (n = 272), 1930 (n = 270) and 1920 (n = 438). Mean age in the general population was 48 years and 52% were women. Data from men and women were pooled and converted into percentages for comparison. Response categories in the self-concept scale range from 1 = strongly disagree, 4 = neither agree nor disagree to 7 = strongly agree. A mean score of 5 on each item can be considered a negative impact on self-concept and we consequently chose a score of 20x5 = 100 and above as cut-off value for high impact on self-concept.

According to the Danish ethical regulation, ethical approval for this study was not needed.

### Statistical analyses

Data analysis was conducted using SAS 9.2 and descriptive statistics was used to summarize Lynch syndrome characteristics with continuous data presented as mean values and standard deviations and discrete data presented as counts and percentages. Univariate analyses were used to explore differences between clinical subsets followed by multivariate linear regression analysis. In the Lynch syndrome cohort the distribution of quartile SOC scores were stratified for age groups, similar to data from the reference population and comparison was made using Fisher’s exact test. This test was also used to compare differences between high and low impact on self-concept including the stratified analysis. Correlations between SOC and self-concept, for the total scale as well as for the sub-scales, used scatter plots and assessment with Pearson correlation coefficient. Student’s t-test was used for comparison of mean values in the two groups.

## Results

**Figure 1:**
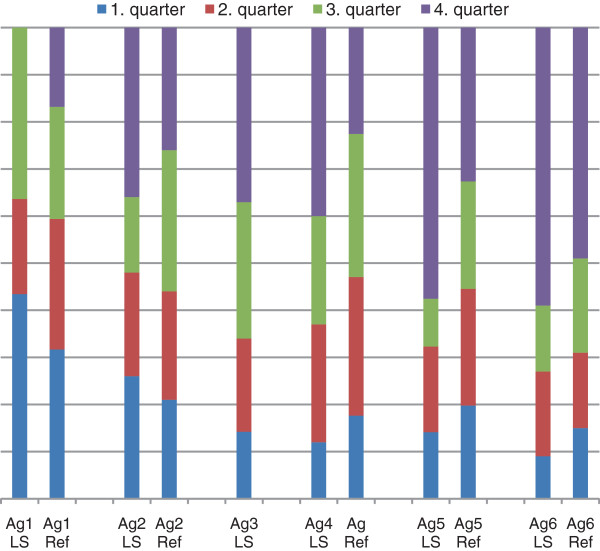
Distribution % of SOC values in the Lynch syndrome cohort and the general population in age groups.

The overall response rate was 80%. Data on both SOC and self-concept were available from 339/345 (98%) informants. The informants had a mean age of 48 years, ranging from 18 to 87 years and included 175 (51%) women and 139 (40%) individuals with a previous diagnosis of cancer. The mean self-concept score was 55 (SD 22.5) ranging from 20–132. The mean SOC value in the Lynch syndrome subset was 70 (SD12) compared to 65 (SD 11) in the general Danish population [38] (p < 0.0001). When the cut-off values (<63/63-73/73-79/> 79) for SOC were applied to the Lynch syndrome cohort, 16% of the mutation carriers reported weak SOC scores, 21% under average, 22% over average and 40% reported high SOC scores. Univariate analyses did not reveal any differences in SOC scores in relation to sex (p = 0.41). Individuals with previous cancer had somewhat higher scores (mean 71 versus 68) (p = 0.047), but this finding was not significant in multivariate analysis adjusting for sex and age (p = 0.3). The distribution of SOC scores in the Lynch syndrome cohort was similar to the general population. Though SOC increased with age in the reference group as well as in the Lynch syndrome cohort, the effect was more pronounced (Pearson correlation coefficient 0.2) among older individuals with Lynch syndrome who reported significantly higher SOC scores (Figure 1).

**Figure 2:**
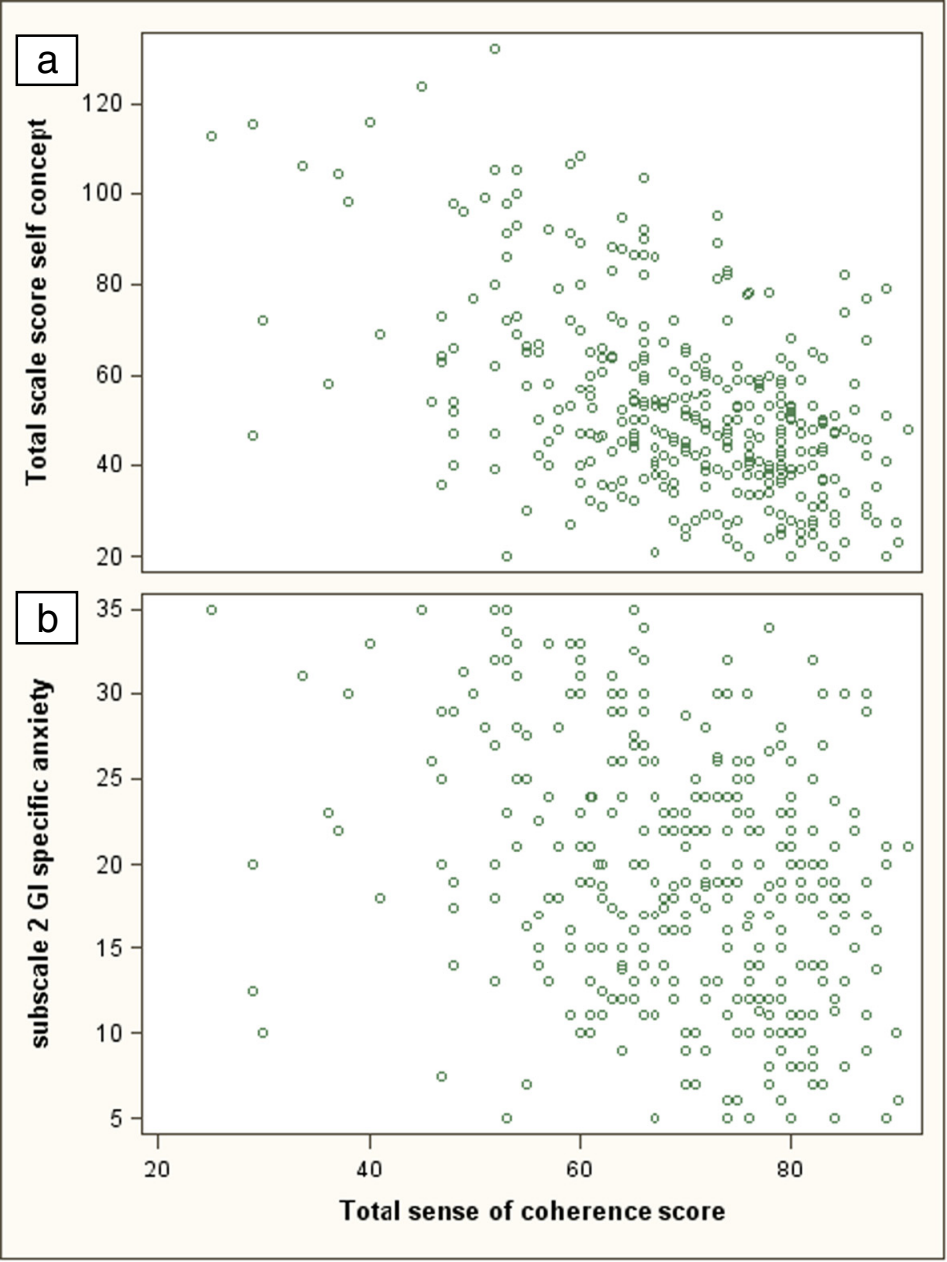
**Correlation between SOC and self-concept scores. a**: SOC and total self-concept scores. **b**: SOC and the Bowel symptom-related anxiety subscale.

SOC and self-concept were correlated, i.e. strong SOC correlated with low impact on self-concept (Pearson correlation coefficient −0.51, p < 0.0001) (Figure 2a). When the SOC scores were correlated to the self-concept subscales, the stigma and vulnerability subscale showed a pattern similar to the total self-concept scale (correlation coefficient −0.54, p < 0.0001), whereas the correlation between SOC and the subscale for bowel symptom-related anxiety was weaker (correlation coefficient - 0.3, p < 0.001) (Figure 2b). In total 4% of the individuals reported high impact on the total self-concept scale, whereas 23% reported high impact on the bowel symptom-related anxiety subscale. Among individuals with weak SOC, high impact on total self-concept was reported by 14% and when the bowel symptom-related anxiety subscale was considered, by 38% (Table 1). When self-concept data were stratified for sex, previous cancer and experiences from cancer in close relatives, women and individuals with experience reported significantly higher scores on the bowel significantly higher scores on the bowel symptom-related subscale.

**Table 1:** **Distribution of SOC and self**-**concept scores**

	**Low impact on**	**High impact on**	
	**self-concept,N (%)**	**self-concept,N (%)**	**P value**
Strong SOC	253 (99.6)	1 (0.4)	<0.0001
Weak SOC	73 (85.9)	12 (14.1)	
	Stigma and vulnerability subscale		
Strong SOC	254 (100)	0 (0)	<0.0001
Weak SOC	75 (88.2)	10 (11.8)	
	Bowel symptom-related subscale		
Strong SOC	208 (81.9)	46 (18.1)	<0.001
Weak SOC	53 (62.4)	32 (37.6)	

## Discussion

Knowledge about how individuals at increased risk of cancer manage life is insufficient, though it would provide an important basis for the development of targeted support. We took advantage of two validated scales, SOC and self-concept, that evaluate different aspects of how individuals handle difficulties and perceive an increased risk of cancer. Whereas SOC represents a more global estimate of how difficult situations are perceived and managed, self-concept relates to the specific impact from hereditary cancer and the need for surveillance. SOC scores in the Lynch syndrome cohort were comparable to those in the general population and in the oldest cohort, mutation carriers indeed reported stronger SOC than age-matched controls (Figure 1). Similar observations, with higher SOC in older individuals at increased risk, have been reported in hereditary breast cancer and in long-term survivors of childhood cancer [39,40]. SOC generally tends to strengthen with increasing age, especially among individuals with strong SOC [30,38,41,42]. Mutation carriers who have reached high age either represent cancer survivors or individuals who despite a high risk did not develop cancer, which may contribute to a perception of having dealt well with a life at increased risk.

SOC and self-concept scores were clearly correlated. Adverse scores on both scales, i.e. weak SOC and high impact on self-concept were only reported by 4%, though when we applied a relatively high cut-off. The subset with adverse scores on both scales most likely reflects a vulnerable subset that may be in need of more general psychological support, though additional data regarding the relation to health status are needed (27;41). Only a smaller group of individuals reported a high impact on self-concept, which supports the notion that the majority of individuals at increased risk do well. When the bowel symptom-related anxiety subscale was considered, weak SOC and a low impact on self-concept were reported by 17% of the individuals. This subset likely represents individuals, who despite less self-reported ability to handle difficult situations, experience a limited impact from the knowledge about hereditary cancer. In colorectal cancer, low SOC has been found to be associated with anxiety and depression after genetic counselling and have been demonstrated to correlate with mental health and lower quality of life [27,32]. Long-term cancer survivors who report a need for support have also been found to have lower SOC scores [40]. Hence, individuals with weak SOC may represent a vulnerable subset despite a minor perceived impact on self-concept. A substantial impact on self-concept related to bowel-related anxiety, despite strong SOC, was reported by 14% and likely includes individuals who generally handle difficult situations well, but find knowledge about hereditary cancer particularly burdening.

## Conclusion

Lynch syndrome mutation carriers report SOC scores comparable to the general population, which suggests that despite an increased risk of cancer, most individuals find themselves capable of handling difficult situations to the same extent as a reference population. When SOC and self-concept data are correlated, different subsets are recognized, which points to diverging effects from learning about hereditary cancer. Further work is needed to determine clinically relevant cut-off values and to assess predictive value in relation to clinical and psychosocial outcomes but the different subsets identified can indicate a need for diversified interventions.

## Competing interests

The authors have declared that there is no conflict of interest.

## Authors’ contributions

HVP designed the study, collected data and drafted the manuscript. SL participated in study design, performed statistical analyses and contributed to the manuscript. CC contributed to study design and the manuscript. MN contributed to study design, supported the project financially and contributed to the manuscript. All authors read and approved of the final manuscript.
